# Image Processing Technique for Improving the Sensitivity of Mechanical Register Water Meters to Very Small Leaks †

**DOI:** 10.3390/s21217251

**Published:** 2021-10-30

**Authors:** Marco Carratù, Salvatore Dello Iacono, Giuseppe Di Leo, Consolatina Liguori, Antonio Pietrosanto

**Affiliations:** Department of Industrial Engineering, University of Salerno, 84084 Fisciano, Italy; sdelloiacono@unisa.it (S.D.I.); gdileo@unisa.it (G.D.L.); tliguori@unisa.it (C.L.); apietrosanto@unisa.it (A.P.)

**Keywords:** water leak detection, IoT, smart water meter, smart city, EMPIR

## Abstract

Discovering very small water leaks at the household level is one of the most challenging goals of smart metering. While many solutions for sudden leakage detection have been proposed to date, the small leaks are still giving researchers a hard time. Even if some devices can be found on the market, their capability to detect a water leakage barely reaches the sensitivity of the employed mechanical water meter, which was not designed for detecting small water leakages. This paper proposes a technique for improving the sensitivity of the mechanical register water meters. By implementing this technique in a suitable electronic add-on device, the improved sensitivity could detect very small leaks. This add-on device continuously acquires the mechanical register’s digital images and, thanks to suitable image processing techniques and metrics, allows very small flows to be detected even if lower than the meter starting flow rate. Experimental tests were performed on two types of mechanical water meters, multijet and piston, whose starting flow rates are 8 L/h and 1 L/h, respectively. Results were very interesting in the leakage range of [1.0, 10.0] L/h for the multijet and even in the range [0.25, 1.00] L/h for the piston meter.

## 1. Introduction

The European Metrology Program for Innovation and Research (EMPIR), according to the European Association of National Metrology Institutes (EURAMET), funded, in 2018, the Joint Research Project (JRP), named 17IND13 Metrowamet—“Metrology for real-world domestic water metering”. This project includes goals to study and test smart metering solutions to determine water leakage detection at the household level. The well-known importance of water for human life is a large reason why water leakage is today considered a severe problem even in small quantities [1,2]. Indeed, for this reason, much research about the issues regarding the development of leakage detection techniques have been recently proposed: in [3] it has been reported feedbacks about the use of Automated Meter Reading (AMR) system to detect leakage in a large-scale demonstration site, which is conducted at the Scientific Campus of the University of Lille; in [4] the water management system based on Wireless Sensor Networks (WSN) has been analyzed; while in [5,6] novel leak detection techniques are proposed. In [7], an in-depth review of the existing techniques for water leakage detection has been conducted. However, while some practical solutions exist in the distribution networks for leakage detection, the latter appears to be more challenging at the household level. At this level, indeed, leakage detection is intertwined with the need for remote reading of water consumptions, thus generating many complications regarding (i) the leak detection algorithms, (ii) the water sensors employed, (iii) the wireless communication technology adopted, and finally, though not less critical is (iv) the electronic consumption of the devices used for the aim.

(i) The daily or weekly users’ consumption profiles are the basis for most leakage detection algorithms developed in recent years. However, a water leak can be sudden or small: while the former generates a significant detachment from the consumption profile, the latter often is submerged in the “floor” of small intakes. For this reason, to develop a new algorithm able to deal with small water leaks, useful metrics must be defined.
(ii) There are two principal categories of water sensors: mechanical or static. Usually, mechanical water sensors are based on pistons or turbines, providing starting flow rates ranging from 1 L/h to 12 L/h. This aspect has a significant impact on the performance of small leakage detection aims. On the other hand, static sensors are mainly electronic devices based on ultrasound. An important aspect is that static sensors provide digital output data, while the mechanical ones run digits and needles on a hardware register.
(iii) To feed the water leaks detection algorithm with valuable data about the phenomena, the water meters must have smart capabilities and be integrated into a communication network. While static meters are electronic digital devices, mechanical water meters need an electronic add-on device to count magnetic pulses or obtain other consumption readings. In addition, the coverage range and bandwidth of the wireless communication channel must be appropriately chosen to satisfy application requirements.
(iv) Long-life batteries must power smart water meters, resulting in weight and dimensions compatible with domestic applications. Data processing and communication features must deal with these rigid constraints.

Logical and functional dependence among the four stages contributes to increasing the complexity of the solution. Some water leakage detectors can be found on the market in the form of high-price (600–1000$) AC-powered autonomous devices that require cutting the pipe to be inserted downstream of the water meter [8,9,10,11]. A battery-powered device is proposed to be added to the plant without cutting the pipe [12], but it works as a consumption monitoring system that sends notifications when consumption exceeds a customer-defined threshold. Generally, good sensitivities (ranging from 0.5 to 5 L/h), but any response time are indicated in datasheets. Due to these characteristics, only solutions that implement suitable small detection algorithms in domestic water meters would spread to the customer as wide as requested by the problem’s importance. This solution is easy to conceive for static water flow meters (ultrasound/electromagnetic) that are fitted with microprocessor-based devices.

However, these devices represent a limited part of the market due to high costs and a not-so-long battery capability. Consequently, leakage detection solutions should also be designed for cheaper mechanical meters representing most domestic water meter markets. For the last 15 years, the authors have been working on low-cost image-based solutions for the remote reading of water consumption measured by mechanical water meters, describing the use of these technologies from the AMR solution based on wireless communication in the Smart Cities field [13,14,15] to the use of the same solution for the development of preliminary water leak detectors [16,17]. The new frontier, towards which today authors want to push the results of their research activities, is the definition of suitable metrics to detect small leakage starting from images of the mechanical register of water meters. According to the literature, most water leakage detection techniques compare consumption thresholds in terms of flow rate [L/h]. However, only the static flow meters are compatible with the latter approach, while it does not fit entirely with volumetric mechanical meters, which measure volume [m^3^].

The continuous offtake and the Period with Null Consumption (PWNC) monitoring represent a practical approach to developing a robust method for detecting small leakages. In more detail, both quantities are time interval values characterized, respectively, by the non-zero or null value of measured flow [L/s]. In the absence of leaks, the offset can be continuous only for a limited time interval (a threshold); the presence of water leakage can be detected if a continuous offset overcomes this threshold. The threshold, to be effective, must be chosen differently by day and night: while maximum offtake occurs during daily hours, no considerable long-term offtake is expected during the nightly hours, requiring a threshold consistently lower. If there is no leakage downstream of the meter, several PWNCs should appear for an entire day. On this basis, leakage detection can be made from continuous monitoring of offtake by algorithms that could be implemented on smart meters and central units. In both cases, meter sensitivity (the minimum value of flow rate able to provide a change in the meter display) determines the minimum leakage that any algorithm will detect. On the other hand, this intrinsic limit of meters also represents the minimum flow rates that can increase the measured customer consumption and its bill.

Due to continuous technological progress, vision-based measurement systems can be inexpensive, small, and very performant. Starting from this consideration, the authors propose a solution based on an artificial vision “add-on device” to monitor small consumption by improving the sensitivity of the widespread mechanical register meters. Furthermore, since most domestic water leakages have flow rates lower than the starting flow rates of most water meters, the sensitivity improvement allows for water leakage algorithms as PWNC to extend their operative range to such flow rates.

The paper will be structured as the following: in the first part, a brief description of the hardware employed, consisting of an electronic add-on device capable of mechanical register images acquisition through a suitable camera; subsequently, a presentation of the image processing technique designed to handle the register images taken by the smart add-on and an introduction of the metrics useful for the aim. Finally, the experimental results are presented and discussed.

## 2. Smart Add-on Device for Classical Mechanical Register

**Figure 1:**
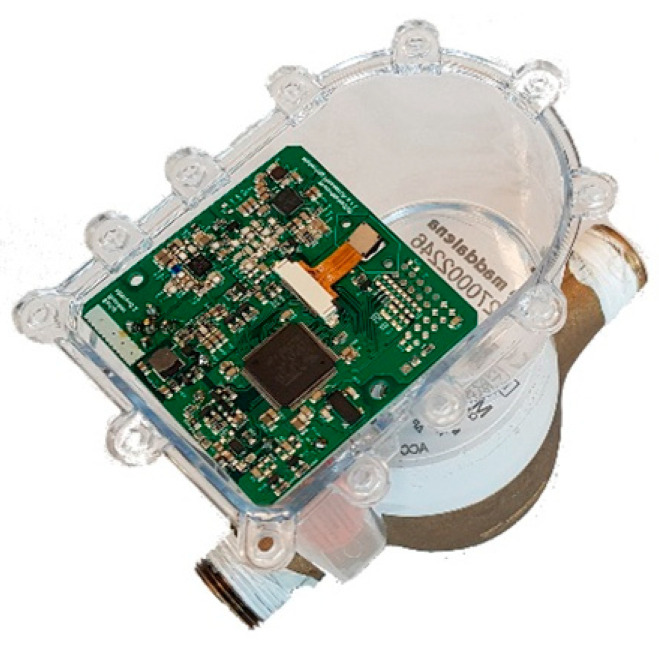
The smart add-on is mounted on a water meter.

The authors designed the “smart add-on” device [18] to provide traditional mechanical counters of smart meters’ typical features, as shown in Figure 1. The smart add-on comprises three main parts: a microcontroller, a short-range communication module, and a camera. The device has been designed for taking pictures of the mechanical register of the water meter with a selectable frame rate, and sending helpful information through the low-range antenna. In addition, to mount the device on most of the mechanical water meters existing on the market.

The characteristics of the main parts composing the add-on are reported in the following:

- The camera module is an OV7670 provided by a low voltage CMOS image sensor. In a tiny footprint, it allows all the functionality of a single-chip image processor and VGA camera. The OV7670 makes available full-frame, sub-sampled, or windowed 8-bit pictures in different formats. The module is controlled via the Serial Camera Control Bus (SCCB) interface. The raw output image can be transferred to a suitable microcontroller using the Digital Camera Module Interface (DCMI). In addition, the consumption of the camera module is low: considering a 30 fps, its current consumption is less than 20 mA at 3 V in the active state and less than 100 μA in standby.
- The processing unit employed is a high-performance microcontroller STM32F4. It belongs to a microcontrollers’ family based on the general-purpose ARM^®^Cortex^®^-M4. The STM32F4 is widely employed in many fields such as entertainment, powertrain, industrial automation, etc. In more detail, the microcontroller is based on a RISC core architecture at 32-bit operating up to 168 MHz. In addition, the Cortex M4 core implements full DSP set instructions and provides a Floating-Point Unit (FPU) single precision. The microcontroller offers, in addition, three 12-bit ADCs, two DACs, one SCCB, and one DCMI. Its memory features include a high-speed Flash memory RAM of up to 1 Mbyte and an SRAM of up to 192 Kbytes.
- The hardware responsible for the low-range communication is in charge of the transceiver CC1120 manufactured by Texas Instruments, with the power front-end module SKY65367-11 manufactured by SKYWORKS. The CC1120 transceiver conforms with EN 13757-4:2013 standard, regulating in a short-range network the WM-Bus operation. In more detail, the prototype has been set up to operate at maximum transfer power in N2a mode. In addition, the SKY65367-11 has been adapted to enhance the power provided to the antenna at up to 27 dBm.
- The smart add-on is battery-powered, utilizing two low discharge lithium batteries at a total capacity of 7000 mAh (3.6 V).

The microcontroller has been suitably programmed in C language, using the Keil uVision software, which allows the compile, program, and debug the C-code for these devices’ typologies.

## 3. Image Processing Techniques and Metrics

According to the PWNC algorithm, an absence of leakage can be assumed when no changes are seen in the water consumption measured by the water meter, according to its metrological characteristics (e.g., starting flow rate, range, and sensitivity). However, due to the most common water meter’s mechanical characteristics adopted in the domestic scenario, a different approach can be pursued for discovering a water leakage. Indeed, these meters characterized by mechanical elements, such as a turbine or piston, if subjected to a long period under a small water flow (smaller than the starting flow rate), will reveal an intermittent movement of the needle. This fact is caused by the accumulated quantity of water, which will cause an inertial force to run the main shaft of the water meter for an instant.

The images acquired by the camera are processed to monitor the consumption and, overall, to detect small leakage [18], characterized by the absence of a period with null consumption. Therefore, the image processing software aims to monitor the counter’s display changes or, in other words, analyze the similarity/dissimilarity between successive images of the counter.

Different approaches can be used to measure the similarity/dissimilarity between images, such as Cross-Correlation, Euclidean Distance, Manhattan Distance, and many others [19,20,21]. However, Cross-Correlation is used in many applications thanks to its non-polarized estimate. Considering two digital images, the 2D cross-correlation coefficient $r\left(I_{A},\;I_{B}\right),$ among two images, *I_A_* (*i*, *j*) and *I_B_* (*i*, *j*) with the same dimension can be evaluated as:

(1) $$r\left(I_{A},I_{B}\right)=\frac{\sum_{j}\sum_{i}\left(I_{A}\left(i,j\right)-\bar{I_{A}}\right)\cdot \left(I_{B}\left(i,j\right)-\bar{I_{B}}\right)}{\sqrt{\sum_{j}\sum_{i}{\left(I_{A}\left(i,j\right)-\bar{I_{A}}\right)}^{2}}\cdot \sqrt{\sum_{j}\sum_{i}{\left(I_{B}\left(i,j\right)-\bar{I_{B}}\right)}^{2}}}$$

where $\bar{\;I_{A}}$ and $\bar{\;I_{B}}$ represent the mean values of the two images *I_A_* (*i*, *j*) and *I_B_* (*i*, *j*).

In more detail, the cross-correlation can fall only in values belonging to the range (−1, 1), where it assumes a value equal to 1 when the two compared elements are identical, while its value lies on −1 when they are opposite; if the elements do not have any correlation, its value becomes 0. Thus, to monitor the meter’s display changings, at each nth new frame acquisition (*I_n_*), the cross-correlation factors can be calculated as:

(2) $$r_{0}(n)=r\left(I_{n},I_{0}\right)$$

where *I*_0_ is the first image acquired (in the future mentioned as reference image) and the *I_n_* is the current acquired image.

**Figure 2:**
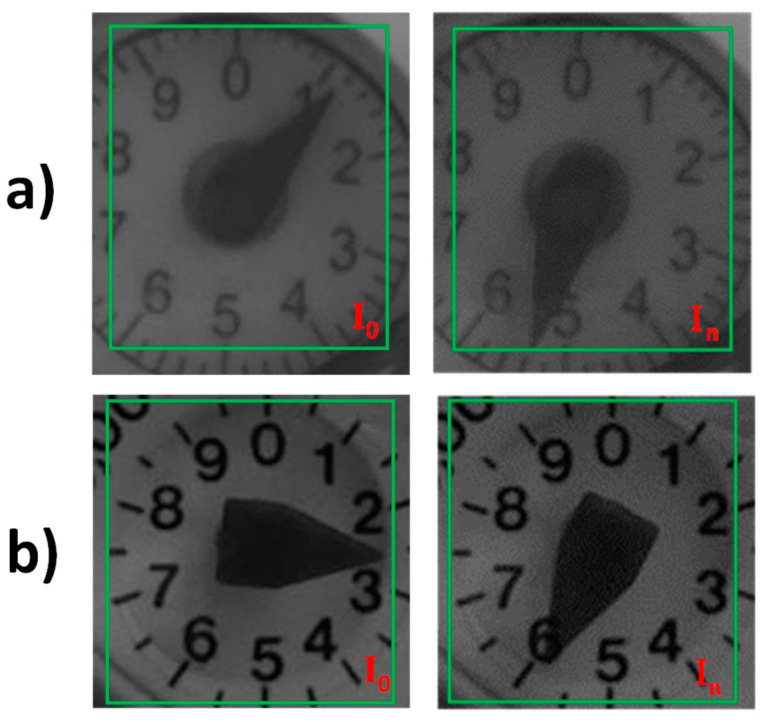
Acquired pictures on both water meters. In (**a**) the volumetric, in (**b**) the multijet; green boxes indicate the ROI where the cross-correlation is evaluated.

Using a PWNC algorithm based on the correlation between two consecutive meter display images allows, using the principle described above, the detection of leakage under the water meter starting flow rate, improving as a consequence, the sensitivity of mechanical registers concerning the leakage phenomena. Indeed, the leakage’s sensitivity corresponds to the water meter sensitivity for all the existing water leakage devices and technology under research. The feasibility and the validation of the method proposed will be reported in the experimental result section, leading to the definition of metrics able to discover leakage, characterized by a flow rate lower than the starting flow rate declared by the manufacturer. As shown in [18], particular consideration should be paid in the definition of the Region of Interest (ROI) on the image where the cross-correlation is applied to have enough sensitivity to a relevant dissimilarity: the correlation factor is an average quantity, and the no-changing parts must not be considered in the calculation. As an example, in Figure 2a,b, three images with two different ROI employed (respectively, needle and digit, highlighted both with green boxes) are reported: the similarity between $I_{n},\;I_{0}$ is quantified with an *r*_0_ = 0.797 for the Figure 2a, and *r*_0_ = 0.765 for Figure 2b. The different results among the calculated indexes highlight the importance of the ROI selection to have enough sensitivity concerning the aim.

In addition, to get information about the presence of a flow rate different from zero, and define metrics to discriminate a “period with null consumption” with flow rates lower than the water meters starting flow rate, experimental tests must be carried in both static and dynamic conditions to discover the metrics of interest.

## 4. The Test Rig

### 4.1. The Measurement System

**Figure 3:**
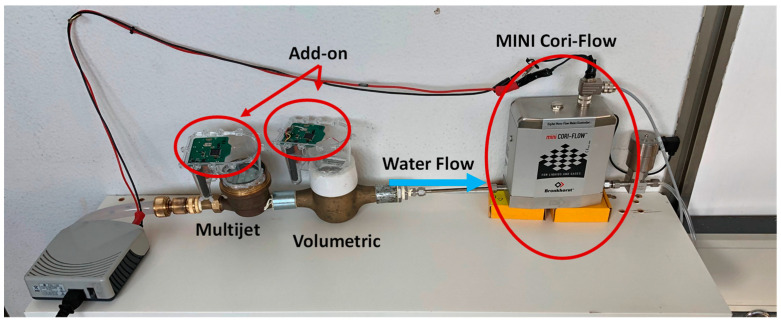
The test rig developed.

The measurement setup has been set in the University of Salerno laboratories, developing a test rig to generate a calibrated constant flow for more than one water meter mounted in series (see Figure 3). It includes a precise and compact instrument to measure and control the water mass flow based on the Coriolis measuring principle: MINI CORI-FLOW™ M15 by Bronkhorst [22]; the metrological performances of the calibrated instrument are reported in Table 1. The instrument can also control and regulate the outcoming water flow, thanks to a valve enabling suitable software to select constant water flows and develop a customizable water consumption profile. The measured values of the selected profiles are stored thanks to the previously mentioned software to be analyzed at the end of the test. Thanks to the test rig feature described above, various tests were carried out at flow rates belonging to small domestic leakages range [0–30] L/h [4].

### 4.2. The Water Meters

The mechanical water meters considered for the scope belong to the two most prevalent domestic water meters groups: multijet and piston. Their principal feature is to measure the total volume of cold water consumed since a reset. The operation principle of the multijet water meters is based on the water flow passage via symmetrical ducts opened in a chamber where a turbine is placed. The latter is run by the water jets impacting on it. As can be figured out, in this way, the turbine’s speed is proportional to the inlet water flow. The rotation of the turbine moves the mechanism of the water meter mechanical register, allowing the measurement of the water volume consumed. The multijet water meter used in this work has five digits and four needles, where the last significant digit corresponds to 1 m^3^, and the last needle indicates 0.0001 m^3^ of resolution. It can be realized that the multijet is one of the most used water meters at the household level, thanks to its simplicity and low cost.

**Figure 4:**
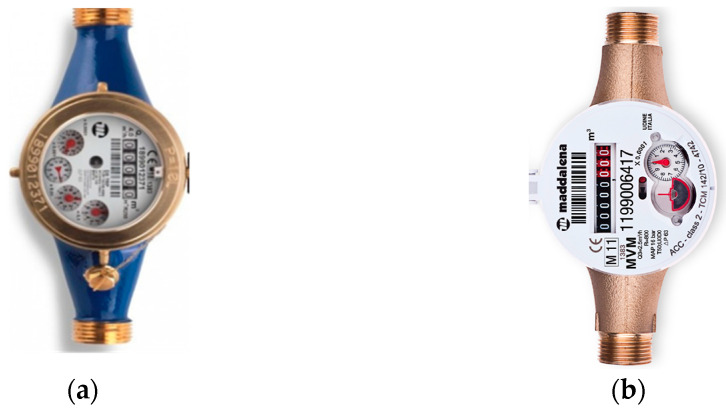
The water meters employed: (**a**) the multijet, (**b**) the volumetric.

On the other hand, the piston water meter assures higher accuracy and sensitivity at low flow rates but has a much higher cost. The operation principle of the piston water meter is represented by the rotation of a piston in a specific volume chamber. A fixed quantity of water will pass through the piston chamber for each rotation. The piston water meter (or more commonly named volumetric) selected for this work presents eight digits and one needle, where the last significant digit is equal to 0.001 m^3^, and the last needle corresponds to 0.0001 m^3^ of resolution. Table 2 reports the nominal hydraulic performance, while Figure 4 shows the two water meters considered for the tests.

## 5. Experimental Results

The idea beyond the proposed leakage detection technique is to find metrics sensitive to the previously described phenomena (intermitting operation of the water meter under the starting flow rate) to improve the whole meter’s sensitivity. Using the test rig described in Section 3, several measurements were made on both water meters. The experimental tests were made first with constant flow rates and then with dynamic water consumption profiles, highlighting the ability of metrics presented later to discover a leakage under the starting flow rate of the water meter. The electronic devices were added to them and were programmed to take 3600 frames/h. The tests were carried out considering external perturbations of the measurement setup, such as vibrations (due to real scenario) or other external parameters influencing the correlation indexes calculation. An actuator made vibrations in the range of 1–100 Hz to emulate real scenarios such as vibrations due to daily traffic [23]. The leak detection technique was mainly tested at the typical operating pressure of water at the household level (i.e., 2 to 4 bar). A few other tests were made under (within 1 bar) and over this range without any problem.

### 5.1. Static Profile

**Figure 5:**
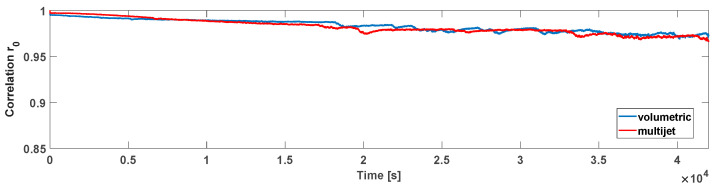
Correlations *r*_0_ with a null water flow condition for both the water meters considered.

**Figure 6:**
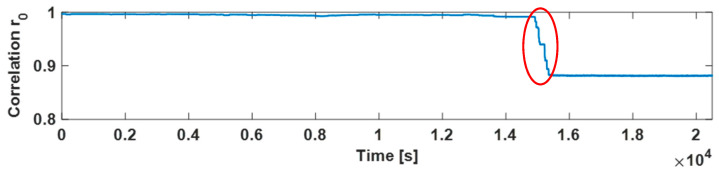
Evolution of *r*_0_ for a flow rate of 1 L/h.

Firstly, the proposed *r*_0_ index (correlation index between the actual image and the reference one) mentioned in Section 3 has been calculated for the water meters considering a null flow rate. This test has led to evaluating the *r*_0_ variability, over an extended observation window, due to everyday external events such as the daily external light changing or the random vibration. With this aim, Figure 5 reports correlations *r*_0_ calculated on the ROIs containing the needles (reported in Figure 2) when a null flow is applied for 12 h. As can be seen over all the periods, the correlations *r*_0_ fall into a slight interval for both meters (respectively, 0.997–0.967 for the multijet and 0.995–0.969 for the volumetric). Then, the same index *r*_0_ has been calculated considering a flow rate of 1 L/h for the multijet water meter (lower than its starting flow rate). As shown in Figure 6, around the fifth hour of the test (see red circles), the accumulated quantity of water showed a drop of the correlation r_0,_ revealing the intermittent movement of the needle as described in Section 3.

For this reason, the *r*_0_ calculation has been revealed to be a valuable metric to be considered for leakage detection purposes. However, the only observation of instantaneous values of *r*_0_ could not be sufficient to provide a reliable leakage indication since unexpected external influences, such as shocks or knocks, could influence the *r*_0_ value.

An effective parameter that could be immune from the issues mentioned above (fast-changing of *r*_0_ in consequence of external events) could be the standard deviation of the *r*_0_ over a sliding window (σ_r0_). The window length selection must be made considering the trade-off between a too-small window that could lead to false detection and a too-long window, presenting lower sensitivity to the phenomena under observation, missing the detection. With this aim, a window of 1000 samples has been revealed to show the best performance, showing an adequate sensitivity according to the phenomena under observation (intermittent operation after a long observation).

**Figure 7:**
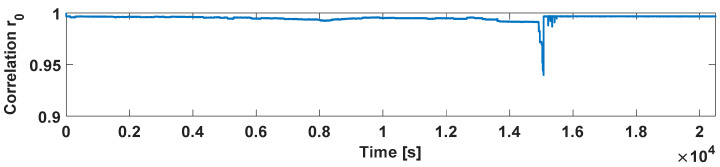
Evolution of *r*_0_ for a flow rate of 1 L/h with the reset strategy enabled.

It has to be pointed out that due to intermitting operation or regular consumption, the actual acquired image can change significantly from the first acquired one, I_0_ (reference image), and consequently, *r*_0_ decreases, reducing the sensitivity to very low consumption. This problem can be handled by replacing the reference image I_0_ in (2) with the current image I_n_ after detecting a consumption as can be seen in Figure 7, where, at this time, after the intermitting phenomena, the correlation *r*_0_ will fall back to the highest value representing a null flow rate condition.

**Figure 8:**
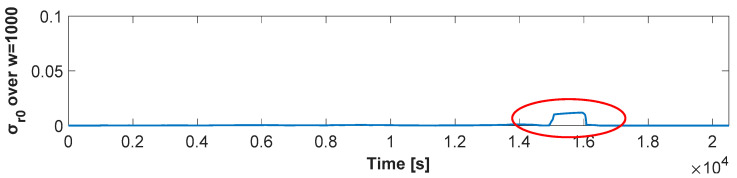
Evolution of σ_r0_ for a flow rate of 1 L/h.

As validation, Figure 8 reports the σ_r0_ calculation for the static profile test of 1 L/h previously presented in Figure 7. As can be seen in the red circle, the σ_r0_ can highlight even better the dropping event. For this reason, the evaluation of the standard deviation σ_r0_ over a sliding window has been revealed to be a metric showing an adequate sensitivity to the leakage detection aim. This analysis shows that, by comparing *r*_0_ and σ_r0_ with suitable thresholds, a PWNC can be identified. With this aim, Table 3 reports the minimum *r*_0_ and the maximum σ_r0_ values evaluated in three different static test profiles: (i) a zero-flow rate profile, (ii) three profiles with flow rates lower than the starting flow rate, and (iii) a profile considering the starting flow rate. Each static test profile has a duration of 6 h, including the actuation of spurious mechanical vibration representing external influences; lower observation periods lead to a wrong characterization of the indexes introduced above since they do not experiment with all the possible real scenarios that can be found on the field, while longer observation windows have shown compatible results with the ones presented in the following. The same analyses can be drawn for both the water meters: as expected, concerning the profile (i), the maximum value of the *r*_0_ and the minimum value of σ_r0_ over the sliding window considered were observed.

Furthermore, concerning the profile (ii), changes in both *r*_0_ and σ_r0_ were experimented representing the ability to detect sporadic phenomena related to the intermitting operation of the water meter in these operating conditions. Finally, regarding profile (iii), the lower *r*_0_ and the highest σ_r0_ were observed concerning all the test profiles. Generally, it can be affirmed that the *r*_0_ values decrease and the σ_r0_ over the sliding window increase significatively for the three-profiles, starting the observation from the zero-flow rate to their relative starting flow rate. Thus, Table 3 shows that both the water meters’ principles allow using metrics proposed by the authors to discover water consumption under their respective starting flow rate, improving their sensitivity concerning the leakage detection operation. Furthermore, the *r*_0_ and σ_r0_ extracted from Table 3 can be used as the threshold for a PWNC algorithm: it can be made by comparing new calculated indexes with those reported in the first line of the table. The latter’s ability allows classical water leakage algorithms to extend their operative range to flow rates much lower than the starting flow rate of the water meter, where they belong in most domestic water leakages.

### 5.2. Dynamic Profile

**Figure 9:**
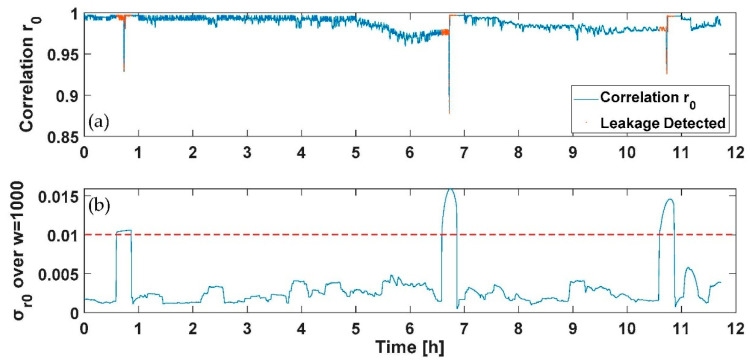
Test on dynamic profile without leakage for the multijet water meter: (**a**) the *r*_0_ correlation and the point detected corresponding to consumption in red, and (**b**) the moving average σ_r0_ and in red the threshold retrieved from Table 3 to discriminate a flow rate different from zero.

**Figure 10:**
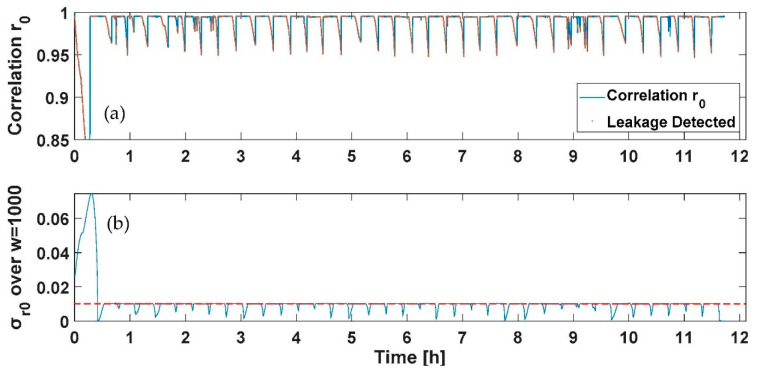
Test on dynamic profile with a continuous leakage for the multijet water meter: (**a**) the *r*_0_ correlation and the point detected corresponding to consumption in red, and (**b**) the moving average σ_r0_ and in red the threshold retrieved from Table 3 to discriminate a flow rate different from zero.

**Figure 11:**
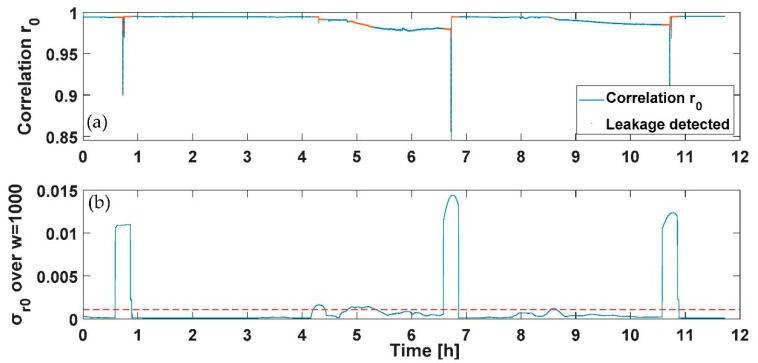
Test on dynamic profile with a continuous leakage for the piston water meter: (**a**) the *r*_0_ correlation and the point detected corresponding to consumption in red, and (**b**) the moving average σ_r0_ and in red the threshold retrieved from Table 3 to discriminate a flow rate different from zero.

**Figure 12:**
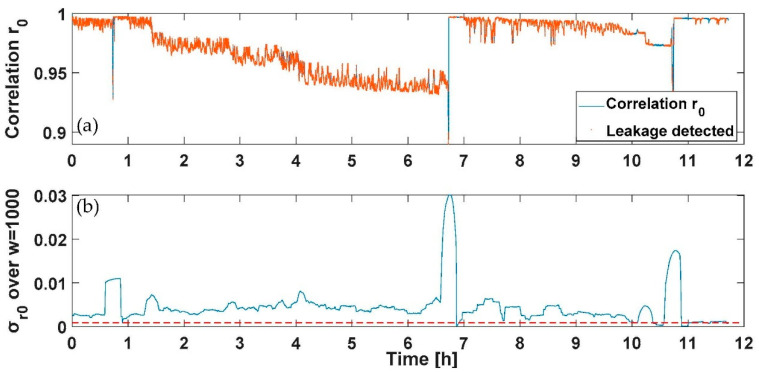
Test on dynamic profile with a continuous leakage for the piston water meter: (**a**) the *r*_0_ correlation and the point detected corresponding to consumption in red, and (**b**) the moving average σ_r0_ and in red the threshold retrieved from Table 3 to discriminate a flow rate different from zero.

To verify the feasibility of the results drawn from the static profile and employ them to design a PWNC based on the electrical add-on presented, nightly dynamic consumption profiles have been fed to the water meters considered (both multijet and piston) with the suitable test rig developed. Specifically, two tests have been conducted with a duration of 12 h; a first profile includes a flow’s variability in the range 0–30 L/h provided three single times during the 12 h test (representing the services used by the user during the night); the three consumptions considered have been selected to represent the use of a domestic utility such as of the toilet or faucet during the night and are characterized by the first consumption of 24 L/h of 5 min introduced after 50 min from the start of the test, the second consumption of 30 L/h of 5 min introduced after 410 min from the start of the test and the last one of 26 L/h of 5 min introduced after 650 min from the start of the test. A second profile was composed of the previously designed profile with the addition of an initial consumption of 30 L/h with a duration of 15 min and a continuous leakage lower than the starting flow rate of the multijet water meter considered (selected in this case of 1 L/h). In Figure 9, Figure 10, Figure 11 and Figure 12, the *r*_0_ and the σ_r0_ drastically change only in correspondence between the three programmed events; according to the previous section analysis, the tests aim to reveal the persistence of conditions in which the σ_r0_ over the sliding window of 1000 samples considered falls in a zone characterized by a flow rate different from zero (over the threshold reported for the first measurable flow rate in Table 3 and reported in red in Figure 9a and Figure 11a). The overlap among consecutive sliding windows has been selected experimentally to the N-1 sample to maximize the index’s responsivity.

Furthermore, the size of the window N has been chosen by evaluating the sensitivity of the σ_r0_ calculated over the window, to the leak phenomena. Considering a too-short window, the sensitivity to the leak of the σ_r0_ calculated over the window is increased, but it is also more influenced by external effects leading to false alarms. A too long window, the opposite is observed: the influence of external effects is decreased, but sensitivity to the leak is too low, resulting in missed alarms. Following this approach, the experimentally chosen window of N = 1000 samples (1000 s) has shown satisfying performance in all the tests reported. Moreover, their value can also give information of the kind of flow rate detected, mainly if it belongs to a flow rate under or over the starting flow rate of the water meter considered.

On the other hand, Figure 10 and Figure 12 report the case of an initial consumption with a sudden leakage for 12 h, as previously mentioned. As for Figure 9a and Figure 11a, the detected points representing a leakage have been reported in red. As shown in Figure 10 and Figure 12, the sudden leakages force, in this case, the indexes calculated to continuously present values indicating a flow rate under the starting flow rate, except for the first negative peak in *r*_0_ (and positive in σ_r0_), which represents a programmed consumption.

## 6. Conclusions

The paper faced the problem of using mechanical register water meters to detect flows lower than the starting flow rate. The solution was found by implementing an image processing technique in an electronic add-on device that continuously acquires mechanical register pictures. The technique relies on some metrics that detect the accumulation phenomena caused in mechanical water meters by small flow rates, even if lower than their starting flow rate. In particular, the paper presented an approach for similarity detection in image processing based on cross-correlation. Since even very small changes in correlated images of the mechanical registers of the water meters provide significant and repetitive variability of the 2D cross-correlation factor, a reliable threshold can be established to distinguish similarity from dissimilarity. Tests suggest a consideration of a 0.1 threshold for the *r*_0_ correlation factor to detect a flow rate higher than 1 L/h for the multijet and 0.25 L/h for the piston. This approach consistently improves the sensitivity of both mechanical register meters in the zero detection, making them more efficient and reliable in identifying PWNC. Furthermore, due to the small computational burden required by the *r*_0_ calculation, the proposed technique can be considered a starting point towards implementing metrics aimed at small water leakage detection in smart meters.

## Figures and Tables

**Table 1:** Technical specification MINI CORI-FLOW™ M15.

**Flow range (intermediate ranges available)**	min. 0.2…5 kg/h; max. 3…300 kg/h
**Mass flow accuracy**	0.2% of rate;
**Repeatability**	Liquid: 0,2% of rate;
**Zero stability (ZS)**	0.5% of rate ± ½(ZS * × 100/actual flow)%
**Response time (sensor)**	≤200 ms
**Temperature accuracy**	on zero: <5 g/h/°C;
**Density accuracy**	on span: <0.001% Rd/°C;

**Table 2:** Hydraulic performance of water meters.

METER	Size [inch]	Starting Flow [L/h]	Q1 [L/h]	Q2 [L/h]	Q3 [L/h]	Q4 [L/h]
Multi-jet	1/2"	7-8	15.6	25	2500	3130
Volumetric	1/2"	1	16	25.6	1600	2000

**Table 3:** Mean *r*_0_ and Maximum Standard Deviation calculated on a sliding window of *r*_0_ of size w = 1000.

Multijet Water Meter				Piston Water Meter			
Flow Rate [L/h]		Min (*r*_0_)	Max(σ r_0w_)	Flow Rate [L/h]		Min (*r*_0_)	Max(σ r_0w_)
Zero Flow Rate	0	0.986	0.0002	Zero Flow Rate	0	0.987	0.0002
Under Starting Flow Rate	1	0.940	0.012	Under Starting Flow Rate	0.25	0.990	0.001
	3	0.950	0.011		0.5	0.986	0.003
	6	0.934	0.011		0.75	0.970	0.003
Starting Flow Rate	8	0.901	0.042	Starting Flow Rate	1	0.960	0.013
Over Starting Flow Rate	10	0.891	0.034	Over Starting Flow Rate	10	0.784	0.080
